# Spatial Gradient Effects of Metal Pollution: Assessing Ecological Risks Through the Lens of Fish Gut Microbiota

**DOI:** 10.3390/jox15040124

**Published:** 2025-08-03

**Authors:** Jin Wei, Yake Li, Yuanyuan Chen, Qian Lin, Lin Zhang

**Affiliations:** 1 Hubei Key Laboratory of Animal Nutrition and Feed Science, School of Animal Science and Nutritional Engineering, Wuhan Polytechnic University, Wuhan 430023, China; weijin@whpu.edu.cn (J.W.);; 2 Fujian Key Laboratory of Special Aquatic Formula Feed (Fujian Tianma Science and Technology Group Co., Ltd.), Fuqing 350300, China; 3 Hubei Engineering Research Center for Protection and Utilization of Special Biological Resources in the Hanjiang River Basin, College of Life Sciences, Jianghan University, Wuhan 430056, China; 4 Yangtze River Fisheries Research Institute, Chinese Academy of Fishery Sciences, Wuhan 430024, China

**Keywords:** heavy metals, surface water pollution, fish tissue accumulation, gut microbiome, ecological risk

## Abstract

This comprehensive study investigates the spatial distribution of metals in surface water, their accumulation in fish tissues, and their impact on the gut microbiome dynamics of fish in the Qi River, Huanggang City, Hubei Province. Three distinct sampling regions were established: the mining area (A), the transition area (B), and the distant area (C). Our results revealed that metal concentrations were highest in the mining area and decreased with increasing distance from it. The bioaccumulation of metals in fish tissues followed the order of gut > brain > muscle, with some concentrations exceeding food safety standards. Analysis of the gut microbiota showed that *Firmicutes* and *Proteobacteria* dominated in the mining area, while *Fusobacteriota* were more prevalent in the distant area. Heavy metal pollution significantly altered the composition and network structure of the gut microbiota, reducing microbial associations and increasing negative correlations. These findings highlight the profound impact of heavy metal pollution on both fish health and the stability of their gut microbiota, underscoring the urgent need for effective pollution control measures to mitigate ecological risks and protect aquatic biodiversity. Future research should focus on long-term monitoring and exploring potential remediation strategies to restore the health of affected ecosystems.

## 1. Introduction

Surface water pollution, especially caused by industrial discharges, mining operations, anthropogenic activities, and the natural erosion of metallic elements, presents serious ecological and public health threats [1]. Toxic metals and metalloids like arsenic (As), cadmium (Cd), chromium (Cr), mercury (Hg), nickel (Ni), and lead (Pb) are especially problematic owing to their harmful effects, environmental persistence, and inability to degrade biologically [2]. These contaminants can bioaccumulate in aquatic ecosystems, particularly in marine life, rendering them among the most dangerous river pollutants [3]. Even nutritionally required elements such as cobalt (Co), copper (Cu), iron (Fe), selenium (Se), manganese (Mn), and zinc (Zn) may exhibit toxicity at elevated levels by promoting oxidative damage through the generation of reactive species that disrupt cellular structures and biomolecules [3]. In contrast, non-essential metallic and metalloid compounds, including As, Cd, Cr, Hg, Ni, and Pb, provide no physiological value and can trigger harmful consequences—such as carcinogenic, mutagenic, and teratogenic outcomes—even in trace quantities [4]. For instance, consuming water contaminated with excessive Pb or As may result in both long-term and immediate toxicity, potentially increasing cancer risks in exposed populations [5].

The contamination of surface waters with metals presents a significant risk to human health, as these pollutants accumulate in living organisms, diminish biodiversity, and interfere with nutrient cycling in ecosystems [6]. People may be exposed to these hazardous substances through skin contact with tainted water, ingestion of contaminated fish or crops irrigated with polluted sources, and consumption of unsafe drinking water. These exposure routes are vital for evaluating risk quotients and indices, which help determine the potential health impacts of metal bioaccumulation [4]. Unlike organic pollutants, metals resist microbial breakdown, leading to prolonged ecological disturbances that may impair aquatic life, including reduced fish survival rates [7]. Consequently, thorough monitoring of metal levels in rivers is crucial for minimizing health hazards and preserving aquatic ecosystem stability.

As apex consumers in aquatic food chains, fish serve as valuable bioindicators for assessing water quality and pollution levels [8]. Due to industrial and domestic wastewater discharge, as well as natural inputs from lakes and rivers, aquatic species are prone to metal bioaccumulation and biomagnification [6]. These contaminants build up in different fish tissues and organs, with varying degrees of concentration amplification. Fish are particularly useful for evaluating metal toxicity due to their widespread distribution, size variability, and role in human diets [9]. They are a key nutritional source, supplying high-quality protein, essential fatty acids, vitamins, and minerals such as selenium [10]. While trace elements like Cu, Fe, Co, and Zn are necessary for fish metabolism, excessive amounts can cause tissue damage and physiological dysfunction [11,12]. Conversely, non-essential metals like Cd, As, Hg, and Pb are toxic even in minute quantities, posing risks to both aquatic life and humans [13]. When absorbed, these metals can induce oxidative stress, cellular damage, and digestive system impairments, as fish lack efficient excretion mechanisms. Research indicates that metal exposure disrupts gut microbiota, which plays a critical role in fish health. Prolonged contamination, for example, has been linked to disease development in carp due to microbial imbalance [14,15].

The gut microbiota serves vital functions in energy homeostasis and immune regulation [16], while also contributing to intestinal development, physiological structure, and nutrient assimilation in fish [17]. Dominant bacterial phyla in the fish gut include *Proteobacteria*, *Firmicutes*, *Bacteroidetes*, *Fusobacteria*, and *Actinobacteria* [18], with *Proteobacteria* typically representing the most prevalent group, followed by *Firmicutes*, *Bacteroidetes*, and *Actinobacteria*. Together, these phyla may comprise over 90% of the microbial population, though their relative abundance fluctuates with environmental factors like habitat conditions and seasonal changes [19]. Microbial composition and density are further modulated by host age, dietary intake, and external environmental parameters, including water temperature [20]. Exposure to environmental contaminants, particularly heavy metals, can trigger substantial shifts in microbial phylogeny, metabolic pathways, and gene regulation [21], disrupting critical biochemical processes involving carbohydrate, lipid, protein, and nucleic acid metabolism. Such disturbances may precipitate dysbiosis and contribute to metabolic dysfunction in host organisms [22].

Elucidating the organizational principles governing microbial communities is fundamental for preserving symbiotic microbiota homeostasis. Community assembly is shaped by environmental filtering, interspecies interactions, dispersal mechanisms, and stochastic processes [23]. Advances in 16S rRNA gene sequencing have revolutionized microbial ecology studies, enabling detailed characterization of diversity, taxonomy, and evolutionary relationships [24]. Considering fish’s importance as aquatic pollution sentinels and metals’ dual effects on piscine health and microbial ecosystems, research must address the complex relationships between metal contamination, tissue bioaccumulation patterns, and microbial community responses. While existing literature documents metals’ ecosystem impacts, particularly their capacity to induce microbial dysbiosis and oxidative damage in aquatic species [3,4], key knowledge gaps persist regarding the following: (1) the precise pathways through which metals modify gut microbiota composition and function, and (2) how these alterations correspond to pollution gradients. Additionally, the biomagnification potential of metals through trophic levels necessitates systematic evaluation of their tissue distribution patterns and associated ecological risks.

This study aims to address these gaps by examining the spatial distribution of metals in surface water and their accumulation in the tissues of *Siniperca chuatsi*, a species known for its high trophic level and significant role in human nutrition, across different pollution gradients. Additionally, we explore the compositional changes in the gut microbiota of these fish in response to varying levels of metal exposure. By integrating environmental, biological, and microbial data, this research provides a holistic understanding of the ecological and health risks associated with metal pollution in aquatic ecosystems. The findings will contribute to the development of effective strategies for mitigating the adverse effects of metals on aquatic biodiversity and human health, while also advancing our knowledge of microbial community assembly under environmental stress.

## 2. Materials and Methods

### 2.1. Study Area

**Figure 1:**
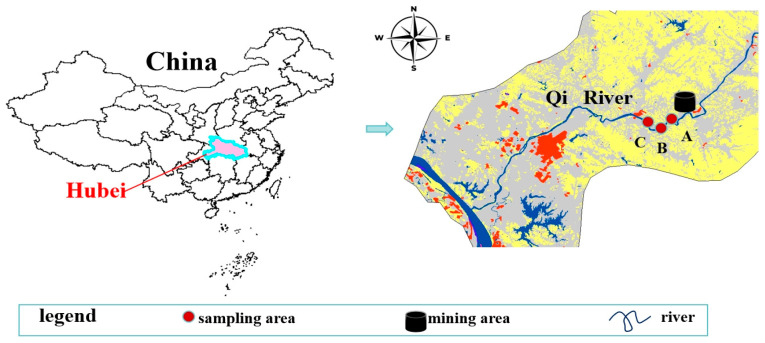
Distribution of sampling areas.

The study was conducted in Huanggang City, Hubei Province, located in the middle reaches of the Yangtze River (Figure 1). The region has a subtropical monsoon climate with an average annual temperature of 17 °C. The sampling area was selected along the Qi River near the Shiguchong mining area in Liuhe Town. Three sampling regions were established along the river: the mining region (A), the transition region (B), and the distant region (C). The distances between the sampling regions are as follows: 249.3 m between A and B, 634.7 m between A and C, and 444.3 m between B and C. Each region included 11 sampling sites (replicates), totaling 33 samples.

### 2.2. Fish Sampling and Analysis

#### 2.2.1. Fish Sampling 

Field-collected specimens of *Siniperca chuatsi* were obtained from each study region, with a minimum of 11 individuals per site. Sample size limitations arose due to the species’ natural scarcity, particularly in heavily disturbed zones such as the mining-impacted Region A and transitional Region B, where anthropogenic pressures have reduced fish populations. Capture was conducted using standard fishing methods, followed by immediate ice preservation to maintain sample integrity during transport to the laboratory. Prior to analysis, specimens were rinsed with distilled water and dissected with ceramic tools to isolate muscle, gut, and brain tissues. Digestion was performed using a Berghof Speedwave Xpert microwave system with a 1:10 (*w*/*v*) ratio of sample to acid mixture (65% HNO_3_ and 30% H_2_O_2_, Merck) [3]. The optimized protocol involved the following: (1) 5 min at 100 °C (1000 W); (2) 10 min at 180 °C (2000 W); (3) 10 min at 220 °C (2000 W); followed by (4) cooling and depressurization. Digested samples were then diluted to 25 mL using deionized water for subsequent analysis.

#### 2.2.2. Determination of Metal Content in Fish Tissues

Metal concentrations in fish tissues were determined using inductively coupled plasma–mass spectrometry (ICP–MS) and inductively coupled plasma optical emission spectrometry (ICP–OES, OPTIMA 8000DV). Both methods were validated in-house and based on standardized protocols, specifically following the guidelines provided by the International Organization for Standardization (ISO) and the United States Environmental Protection Agency (EPA) methods. The detection limits for each metal were determined using the 3σ method, ranging from 0.01 to 0.1 µg/L for ICP–MS and from 0.1 to 1.0 µg/L for ICP–OES. Precision was assessed by analyzing replicate samples (*n* = 6) of a standard solution, with relative standard deviation (RSD) values below 10% for all metals. Recovery tests were performed by spiking known concentrations of metals into blank samples, with recovery rates ranging from 90% to 110%. These validation parameters ensure the accuracy and reliability of our analytical methods.

### 2.3. Water Sampling and Analysis

#### 2.3.1. Water Sample Collection

A Plexiglas water sampler was used to collect water from different depths, which was then mixed and transferred into sampling bottles. Five bottles were collected from each sampling site. Samples intended for metal analysis were immediately acidified with 5 mL of 95% nitric acid. All samples were stored in a portable cooler at 4 °C and transported to the laboratory as soon as possible. To capture the total metal load, including particulate-bound metals, water samples intended for metal analysis were not filtered. Instead, these samples were subjected to a digestion step using nitric acid and microwave-assisted digestion following EPA Method 3015A. This method ensures that all forms of metals are fully dissolved and available for accurate quantification by ICP-MS and ICP-OES. For other analyses, such as total nitrogen (TN) and total phosphorus (TP), water samples were filtered through 0.45-μm filters to remove particulate matter.

#### 2.3.2. Determination of Environmental Parameters

Water quality parameters were analyzed in situ using a YSI Professional Plus multiparameter probe. Ion chromatography was employed to determine NO_2_^−^–N, NO_3_^−^–N, PO_4_^3−^, and SO_4_^2−^ concentrations. Total nitrogen (TN) analysis followed alkaline persulfate digestion, while dissolved inorganic nitrogen (DIN) components (NH_4_^+^–N, NO_3_^−^–N, and NO_2_^−^–N) were quantified using Zhang et al.’s methodology [25]. Phosphorus species were assessed through the ammonium molybdate method for both total phosphorus (TP) and soluble reactive phosphorus (SRP) [26]. Chemical oxygen demand was evaluated via the permanganate index (COD(Mn)). The total dissolved solids (TDS) were determined gravimetrically by filtering the samples through a 0.45 µm filter and subsequent evaporation at 108 °C, following the standardized internal protocols [27]. A summary of the water analysis parameters, including the specific methods and references used for each parameter, is provided in Table S1.

### 2.4. Environmental Risk Calculations

The ecological risk index (Ri) was calculated to assess the potential risks of heavy metals using the following Equations:

R_I_ = ∑E^i^_r_

E^i^_r_ = T^i^_r_ × C^i^_f_

C^i^_f_ = C^i^_0_/C^i^_n_

where R_I_ is the sum of risk factors for all heavy metals at the sampling site, E^i^_r_ is the single potential ecological risk factor, T^i^_r_ is the toxicity response factor for specific metals, C^i^_f_ is the pollution factor, C^i^_0_ is the metal concentration at the sampling site, and C^i^_n_ is the background reference value of heavy metals. The R_I_ risk assessment levels are provided in Tables S2 and S3 [28]. The Nemerow comprehensive pollution index (Pi) was used to represent the overall pollution level at each site. This index is a multifactor weighted environmental pollution assessment index [29], calculated as follows:

$$\mathrm{Pi}\;=\;\sqrt{\left(Imax^{2}+{Iave}^{2}\right)∕2}$$

where Pi is the comprehensive pollution index for each site, I_max_ is the maximum factor index among all samples, and I_ave_ is the average value of the factor indices.

### 2.5. DNA Extraction and PCR Amplification

Gut samples were collected from 11 individuals per sampling region (total of 33 samples) for high-throughput sequencing. Samples were transferred into 2-mL autoclaved tubes containing phosphate-buffered saline (PBS; pH = 7.4) and stored at −80 °C for subsequent use [30]. DNA was extracted using the DNeasy Blood & Tissue kit (Illumina, San Diego, CA, USA) according to the manufacturer’s instructions. The extracted DNA was evaluated for concentration and purity by 1% agarose gel electrophoresis and OD260/280 measurement using NanoDrop 2000 (Thermo Fisher Scientific, Waltham, MA, USA). The V3 and V4 regions of the bacterial 16S rRNA gene were targeted for amplification using an ABI 2720 PCR Thermal Cycler (Thermo Fisher Scientific, Waltham, MA, USA) with the primers 341F (5’-CCTACGGGNGGCWGCAG-3’) and 805R (5’-GACTACHVGGGTATCTAATCC-3’). PCR products were purified using Agencourt AMPure XP beads (Beckman Coulter, Brea, CA, USA), quantified using an Invitrogen Qubit 3.0 fluorometer (Thermo Fisher Scientific, Waltham, MA, USA), and quality-checked using the Agilent 2100 Bioanalyzer (Agilent Technologies, Santa Clara, CA, USA).

### 2.6. Illumina MiSeq Sequencing and Data Processing

Purified amplicons were sequenced using the Illumina MiSeq platform (Illumina, USA) with a paired-end strategy (2 × 250 bp) by Shanghai Majorbio Bio-Pharm Technology Co., Ltd. (Shanghai, China). Low-quality sequences (Q < 20) were filtered using TrimGalore, and reads were merged using FLASH 2. Sequences were clustered into operational taxonomic units (OTUs) at a 97% similarity threshold using the UPARSE program. Representative sequences of each OTU were compared against the 16S rRNA database using mothur.

### 2.7. Statistical Analysis

LEfSe analysis was used to identify differentially abundant taxa between groups (LDA score > 4) [31]. Non-metric multidimensional scaling (NMDS) based on Bray–Curtis distance was employed to analyze the β-diversity patterns of the gut microbiota. PERMANOVA was used to test significant differences in gut microbiota composition between groups [32]. The contribution of stochastic processes to gut microbiota assembly was assessed using a neutral model [33]. Co-occurrence networks were constructed using the random matrix theory (RMT) approach to avoid biases from rare taxa [34]. Only OTUs present in more than six samples were retained for network analysis. Other analyses were performed on the Megi Gene Technology platform. Statistical analysis was conducted using GraphPad Prism 8 (version 8.0.1). To assess these differences, we performed statistical comparisons using one-way ANOVA for normally distributed data and the Kruskal–Wallis test, followed by Dunn’s post-hoc test for non-normally distributed data.

## 3. Results

### 3.1. Environmental Parameters and Characteristics of Metal Pollution

**Figure 2:**
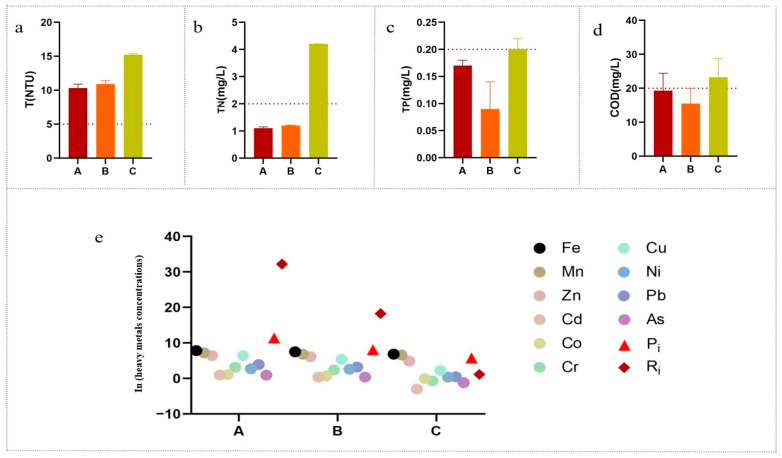
Spatial variation in NTU (**a**), TN concentration (**b**), TP concentration (**c**), and COD value (**d**), in water at different sites, and the variation and comparison of metal and metalloid concentrations (µg/L) (**e**) of different sampling areas (A, B, C). Detection limits: As = 0.001 µg/L, Pb = 0.001 µg/L, Ni = 0.001 µg/L, Cu = 0.01 µg/L, Cr = 0.001 µg/L, Co = 0.001 µg/L, Cd = 0.001 µg/L, Zn = 0.1 µg/L, Mn = 0.1 µg/L, Fe = 0.1 µg/L. The red dashed line represents the Class III standard of China’s “Surface Water Environmental Quality Standard” (GB3838-2002) [35].

In the study area, water quality parameters varied significantly across different sampling sites (Table S4). These variations exhibited a certain spatial pattern. Turbidity (NTU) concentrations at all sampling sites exceeded the maximum allowable concentration by two to three times (Table S4 and Figure 2a). This poses a significant concern for drinking water quality. Samples from region C had the highest turbidity (T = 15.2 NTU), which decreased with increasing distance from the tailings dam (Table S4 and Figure 2a). In region C, concentrations of total nitrogen (TN), total phosphorus (TP), and chemical oxygen demand (COD) also exceeded the maximum allowable concentrations, with TN increasing gradually across the spatial gradient (Table S4 and Figure 2a).

Metal concentrations at different sites are presented in Table S4, and their comparative analysis is shown in Figure 2e as a scatter plot. Prior to analysis, metal concentrations were log-transformed (ln(x)) to improve homoscedasticity and normality. Spatially, region A samples exhibited relatively higher concentrations of various metals and metalloids, which decreased with increasing distance from the mining area. The pollution assessment results also followed this pattern (Table S4, Figure 2b). The Pi decreased across the spatial gradient (Figure 2b). The RI showed a similar trend, with higher values in region A (RI = 32.21) (Table S4, Figure 2e), but still below the low-risk threshold of 40. Overall, region A had issues with T, Fe, Pb, and Mn (Table S4), while region B was mainly affected by T, Fe, and Mn pollution (Figure 2a,e). Region C exceeded standards for T, Fe, Mn, TN, TP, and COD (Figure 2a–e).

### 3.2. Metal Content in Fish Tissues

In this study, we examined the distribution of metals and metalloids within *Siniperca chuatsi* collected from sites A, B, and C (Table 1). Specifically, we measured the concentrations of calcium, magnesium, iron, manganese, zinc, copper, cobalt, nickel, chromium, cadmium, lead, and arsenic in the guts, brain, and muscle of the fish. The results revealed significant differences in metal content across various tissues. For instance, the highest concentration of Cd was found in the guts of fish from site A, reaching 217.7 ± 48.7 μg/kg, while the highest concentration of Pb was observed in the guts of fish from site B, at 809.8 ± 444.1 μg/kg. Additionally, the highest concentration of Zn was noted in the guts of fish from site C, at 276.5 ± 58.9 μg/kg. The range of Mn was 0.6–2.1 mg/kg.

All measured As concentrations in fish tissues (24.9–287.1 μg/kg) remained below the permissible limit of 1.0 mg/kg, with gut tissues showing the highest accumulation, followed by brain and muscle. Copper (Cu) levels ranged from 1987.7–10,069.5 μg/kg, exhibiting a similar tissue distribution pattern (gut > brain > muscle), with muscle tissues from site C containing the lowest Cu concentrations (Table 1). Ni was predominantly concentrated in gut tissues (1478.2–1895.2 μg/kg), while chromium Cr showed the highest accumulation in gut samples (1995.2–10,041.3 μg/kg). Co concentrations (110.9–654.4 μg/kg) peaked in tissues from site A, whereas Zn levels (19.1–276.5 μg/kg) were highest in the intestinal tissue of sample C. Pb concentrations (67.6–809.8 μg/kg) reached their maximum in sample B.

### 3.3. Gut Microbiota Composition and Assembly Mechanisms of Siniperca chuatsi Along a Metal-Pollution Gradient

**Figure 3:**
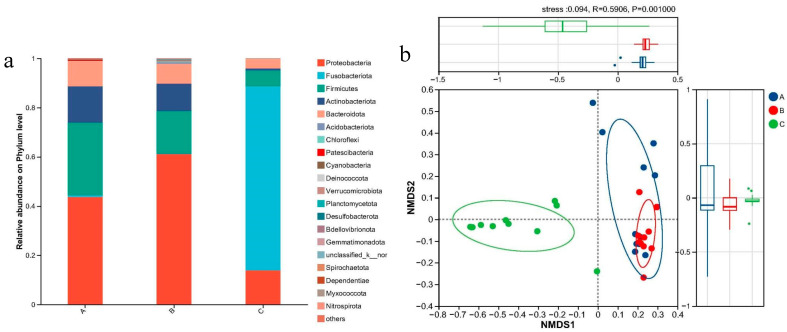
Bacterial community composition with relative abundance >5% at the phylum level by different sampling areas (A, B, C) (**a**). Non-metric multidimensional scaling (NMDS) plot showing the clustering of gut microbiota (**b**).

The composition and assembly of the gut microbiota in *Siniperca chuatsi* from sampling sites A and B were predominantly characterized by *Proteobacteria* and *Firmicutes*, with relative abundances of approximately 0.8. In contrast, *Fusobacteriota* and *Proteobacteria* were the dominant phyla in the gut microbiota of fish from site C, with a relative abundance of about 0.9. *Acidobacteriota*, *Chloroflexi*, *Crenarchaeota*, and *Planctomycetota* were among the less abundant bacterial phyla across all samples, with relatively low abundances (Figure 3a). NMDS and PERMANOVA analyses revealed significant differences in community composition among the gut microbiotas (Figure 3b), with PERMANOVA indicating *p* < 0.001. Notably, gut microbiotas from sites A and B exhibited higher similarity, whereas site C’s microbiota was distinctly different.

**Figure 4:**
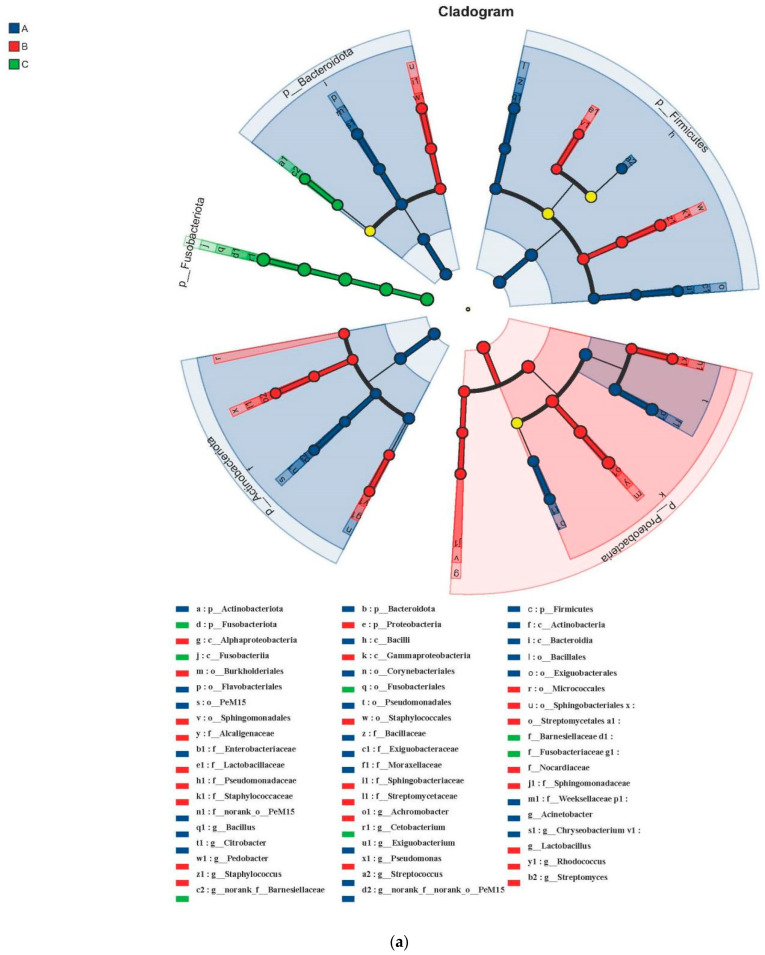

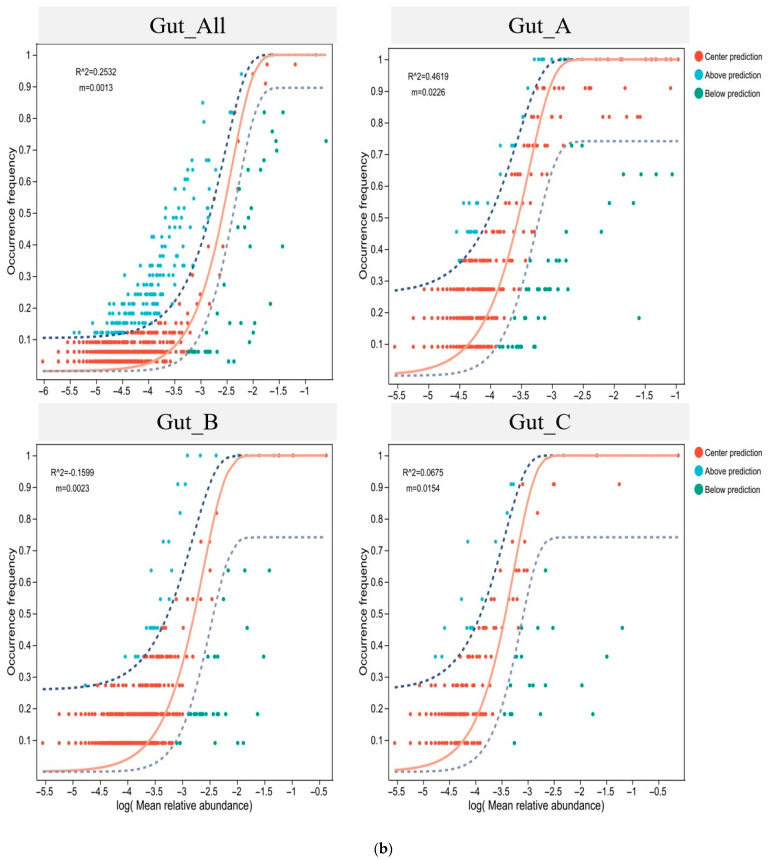
Linear discriminant analysis effect size (LEfSe) showing the clustering of gut microbiota by different regions (A, B, and C) (**a**). The fitness of the gut microbiota to the neutral model (**b**). OTUs that occur more frequently than predicted by the model are shown in red, while those that occur less frequently than predicted are shown in green. Dashed lines represent 95% confidence intervals around the model prediction (blue line).

LEfSe analysis showed significant changes in the gut microbial community structure across the three groups (A, B, and C). Specifically, the relative abundances of the following microbial taxa were significantly increased (LDA score > 4) (Figure 4a). In A, *Pseudomonadales*, *Weeksellaceae*, *Corynebacteriales*, *Flavobacteriales*, *Actinobacteria*, *Actinobacteriota*, *Bacilli*, *Firmicutes*, *Acinetobacter*, *Moraxellaceae*; in B, *Proteobacteria*, *Burkholderiales*, *Alcaligenaceae*, *Achromobacter*, *Gammaproteobacteria*, *Alphaproteobacteria*, *Sphingobacteriales*, *Sphingobacteriaceae*, *Staphylococcus*, *Staphylococcaceae*; and in C, *Cetobacterium*, *Fusobacteriaceae*, *Fusobacteriales*, *Fusobacteriota*, *Fusobacteriia*, *Barnesiellaceae*. Neutral model analysis suggested that the explanatory power of the model was limited for both the combined analysis of microbial OTUs from all sampling sites and the separate analysis of each sampling area, indicating a potential need for further optimization or consideration of additional influencing factors (Figure 4b).

**Figure 5:**
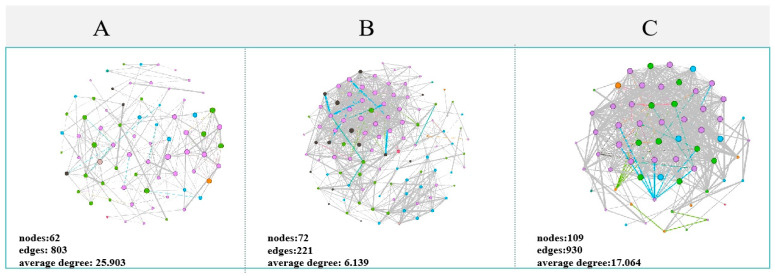
Co-occurrence network showing interaction patterns and correlations of gut microbiota OTUs at the phylum level under different regions (**A**–**C**) (r > 0.6, *p* < 0.05). The size of the nodes is proportional to the betweenness centrality. Edges are colored according to interaction type: red indicates positive correlations, blue indicates negative correlations. The numbers at the bottom left of the network figure are the basic information of the network.

Subsequently, we analyzed how the associations among microbes in the gut of *Siniperca chuatsi* varied with changes in metal pollution. Severe pollution reduced the correlations within microbial communities, with the microbial network in region A having a higher number of nodes (A, B, and C: 62 vs. 72 vs. 109), edges (803 vs. 221 vs. 930), and average degree (25.903 vs. 6.139 vs. 17.064) compared to the microbial networks in regions B and C (Figure 5 and Table S5). In comparison, environments with lower metal pollution exhibited denser networks. Metal pollution also altered the patterns of bacterial interactions within the gut of *Siniperca chuatsi.* With increasing pollution, the occurrence of negative correlations among microbes gradually increased.

## 4. Discussion

### 4.1. Water Chemistry

According to the Surface Water Environmental Quality Standards (GB3838-2002) [35] Class III criteria, turbidity (NTU) at all sampling sites exceeded the maximum permissible concentration by two to three times (Table S4 and Figure 2a). This raises significant concerns regarding drinking water quality. The elevated turbidity is likely attributable to soil particles, which constitute a substantial portion of suspended solids. Region C exhibited the highest turbidity levels, indicating a greater presence of colloidal organic particles, silt, plankton, clay, and other suspended solids [36]. Turbidity values exceeding 5.0 NTU can adversely affect fish gills and photosynthetic activities [37]. The presence of clay, silt, organic matter, plankton, algae, metals (e.g., lead, cadmium, and mercury), organic pollutants, and microorganisms in aquatic habitats can contribute to increased turbidity, potentially leading to adverse health effects and sediment accumulation [37].

The pollution impact of tailings pond leakage and its diffusion into downstream habitats has been well-documented in previous studies [38]. Water contamination primarily stems from high potential energy gradients, which enhance leakage rates into surrounding groundwater systems. In this study, the Pi and the Ri were employed to collectively assess the regional pollution levels in habitats downstream of the tailings pond. Spatially, we observed that as the spatial scale expanded outward from the pond area (A), both Pi and Ri values exhibited a declining trend. The mobility of different metals in the environment displayed varying sensitivities to spatial scale changes. For instance, the higher mobility of Ni and Cd facilitated their accumulation in downstream habitats [1]. Tailings pond contamination also resulted in lower pH levels and elevated electrical conductivity (Table S4). TN concentrations in the mining area were lower than those in downstream habitats, likely due to secondary pollution from agricultural activities, such as the discharge of feed from surrounding mandarin fish farming. Although TN levels in the mining area were lower than downstream, they still exceeded the Class III standards of the Surface Water Environmental Quality Standards (GB3838-2002) [35]. This is attributed to increased nitrate ions resulting from mining activities [39]. Furthermore, the diffusion of tailings pond pollution can occur through evaporation, rainfall infiltration, and dust deposition [40]. Seasonal fluctuations in groundwater levels and plume behavior may also influence the subsequent migration of leakage contaminants [41].

### 4.2. Variability in Metal Bioaccumulation in Fish Tissues

The bioaccumulation of metals in fish tissues is influenced by a multitude of factors, including species, age, size, sex, body morphology, diet, tissue composition, physiological traits, feeding behavior, migration patterns, feeding rates, foraging habits, growth rates, reproductive cycles, trophic position, elimination kinetics, and the degree of contamination in the study area [42]. Additionally, metal bioavailability, interactions, aqueous metal concentrations, environmental parameters (e.g., water temperature, salinity), and seasonal variations play significant roles [43].

Fish collected from the study area’s rivers were analyzed for metal concentrations across different tissues. Generally, the bioaccumulation order of metals and metalloids in fish tissues was gut > brain > muscle (Table 1). Elevated metal levels in the gut often reflect high metal content in the diet and low absorption rates, attributed to the low bioavailability of ingested metals. While Cd and Pb are non-essential elements for fish metabolism, Fe, Cu, Zn, Mn, and Co are essential but can pose health risks when their concentrations exceed permissible limits. Fish typically accumulate Mn, Co, and Ni through ion exchange across the lipid membranes of their gills. In this study, guts and brain samples of *Siniperca chuatsi* from all sampling sites exhibited relatively high Fe levels, exceeding the permissible limit of 100 mg/kg. Excessive consumption of fish heads and guts may lead to Fe overexposure. Fe is crucial for maintaining fundamental metabolic processes in fish, while Mn, an essential element for aquatic organisms, can cause severe skeletal and reproductive abnormalities if deficient. Although reducing conditions may influence Mn bioavailability, their effects on fish remain understudied.

Particularly in its inorganic form, As is known to exert carcinogenic effects on fish, potentially leading to genotoxicity, chromosomal breaks, inhibition of DNA repair systems, and delayed mitotic division. As toxicity may also induce oxidative stress, damaging DNA and cells, ultimately resulting in cell death. In most sites, Cu concentrations were highest in fish kidneys, followed by the guts, gills, and liver. Elevated Cu levels in water can impair branchial function, reducing oxygen uptake and compromising predator avoidance and larval development [12]. Although Cu is essential for hemoglobin synthesis and enzymatic processes, excessive levels may cause morphological and histological changes in the liver [11]. In this study, all Cu concentrations were below the maximum limits set by the FAO (1983) [44] and WHO (1993) (30 mg/kg) [45]. Cu is a vital trace element involved in protein synthesis and chlorophyll formation. Reactive toxic metals (e.g., Cr, Cu, Fe) undergo redox cycling, while inert toxic metals (e.g., Hg, Cd, Pb) covalently bind to thiol groups of major antioxidant enzymes [7]. The primary source of Cu in the study area is industrial activities, including mining operations.

Ni, a natural component of the Earth’s crust, is hypothesized to act as a cofactor in Fe absorption [46]. High Ni levels can impair fish immune systems, alter enzymatic activities, and hinder reproduction, with effects varying by species and life stage [12]. Elevated Ni concentrations are often linked to industrial wastewater discharge into natural water systems [46]. In this study, Cr levels in the guts of fish from sampling site B exceeded the FAO threshold of 1.0 mg/kg [44]. Cr toxicity, which is pH-dependent, can lead to behavioral changes, reduced metabolism, and immune system damage. Cr plays a critical role in glucose, lipid, and protein metabolism. Fish are often used as bioindicators in aquatic ecosystems due to their ability to accumulate Cr through dietary intake or ion exchange across gill membranes [47]. Excessive Cr intake has been associated with irregular swimming patterns, inhibited feeding behavior, gill tissue hypertrophy, paralysis, and reduced immune function [12]. High Cd concentrations pose significant risks to aquatic species, causing renal and hepatic damage, behavioral alterations, growth retardation, spinal deformities, and reduced reproductive capacity [11,12]. These findings underscore the complex interplay of environmental and biological factors in metal bioaccumulation and their ecological consequences.

### 4.3. Instability of the Gut Microbiota Network in Siniperca Chuatsi Caused by Metal Pollution

#### 4.3.1. Community Composition and Assembly of the Gut Microbiota

The gut microbiota is essential for maintaining the health, growth, and development of organisms, and its balance can be disrupted by various environmental factors [48]. This makes it a significant focus in environmental assessment studies. A reduction in the diversity and abundance of commensal gut microbiota can impair the intestinal barrier and negatively affect lifespan. The gut microbiota of fish primarily consists of aerobic/facultative anaerobic microorganisms and obligate anaerobes, particularly *Cetobacterium*, *Bacteroidaceae*, *Aeromonas*, and *Clostridium* [49]. In this study, the phylum-level diversity in the gut microbiota of *Siniperca chuatsi* from three sampling regions remained relatively stable, but significant shifts were observed in the relative abundance of phyla in the A and B basins compared to the C basin (Figure 3a).

The C basin exhibited a higher proportion of the phylum *Fusobacteriota*, consistent with previous studies on common carp (*Cyprinus carpio*) exposed to multiple metals [15]. In the A and B basin groups, *Proteobacteria* and *Firmicutes* dominated, likely due to the relatively lower abundance of gut microbiota in these regions (Figure 5). This observation suggests that *Firmicutes* may be the most tolerant taxon under extreme metal concentrations, while other taxa have limited tolerance [50]. *Firmicutes* are known for their carbohydrate fermentation capabilities, producing butyrate, which provides nutrition and energy to epithelial and gastrointestinal cells, enhances mucus production, and functions as an anti-carcinogenic and anti-inflammatory agent. Depletion of *Firmicutes* can impair gut barrier integrity [51]. Thus, the high proportion of *Firmicutes* (Figure 3a) may indicate the fish’s need to maintain health by preserving intestinal barrier function. Meanwhile, *Bacteroidetes* are involved in nutrient absorption and the maturation and maintenance of epithelial cells [16]. In the C basin, *Cetobacterium*, *Fusobacteriaceae*, *Fusobacteriales*, *Fusobacteriota*, *Fusobacteriia*, and *Barnesiellaceae* dominated the gut of mandarin fish (Figure 4a). In contrast, studies on Nile tilapia have shown that *Bacteroidetes* and *Proteobacteria* dominate under cadmium exposure [52]. This suggests that the observed changes in gut microbiota in this study were primarily driven by chromium, which is predominantly absorbed by fish (Table 1).

Given the involvement of the fish gut in digestion, metabolism, and immune responses [53], changes in environmental parameters can alter the composition of fish gut microbiota, as confirmed by NMDS and PERMANOVA analyses (Figure 3b, PERMANOVA, *p* < 0.001). In this study, neutral model analysis indicated that stochastic processes could explain 46.19%, 15.99%, and 6.75% of the variation in gut microbiota assembly in the A, B, and C basins, respectively (Figure 4b). This suggests that stochastic processes may not play a dominant role in shaping the gut microbiota of fish. Notably, the strength of R^2^ and the microbial migration rate (m) varied across basins, with the highest migration rate observed in the A basin (Figure 4b). This implies that metal pollution increases the likelihood of microbial dispersal compared to normal environmental conditions. Previous studies have shown that over 20% of gut microbiota can originate from gill microbiota when fish are grown in natural environments [54]. This phenomenon may be attributed to the disruption of gut microbiota homeostasis by pollutant exposure, providing opportunities for exogenous microbes to invade and colonize the fish gut [55].

#### 4.3.2. Microbial Networks in the Gut

Microbial interactions, including predation, competition, mutualism, commensalism, parasitism, neutralism, and amensalism, play a vital role in maintaining the health and function of both host-associated [56] and free-living microbial communities [57]. For instance, high-fat diets have been shown to reduce the complexity of the gut microbiota network in rice field eels (*Monopterus albus*), characterized by decreased average connectivity, negative associations, and the number of keystone taxa [58]. In this study, significant changes in the co-occurrence patterns of gut microbiota were observed across sampling regions with varying degrees of metal pollution (Figure 3b and Figure 5). High network complexity is a key factor in maintaining microbial community stability under external disturbances [57].

Keystone taxa—functioning as network hubs, connectors, and module hubs—play crucial roles in microbial co-occurrence networks through their extensive intra- and inter-modular connections [59]. Ecological research has demonstrated their dual importance in maintaining network stability and driving biogeochemical cycles of carbon, nitrogen, and phosphorus [60]. Our pollution gradient analysis revealed significant network simplification (*p* < 0.01), manifested through reduced edge numbers. This reflects two key mechanisms: (1) direct metal toxicity from tailing seepage suppressing bacterial activity (e.g., Cetobacterium extinction in Area A), and (2) pollution-induced community shifts favoring tolerant taxa like Pseudomonadales in heavily contaminated zones (Area A), consistent with prior observations [61].

## 5. Conclusions

By analyzing metal concentrations in water and fish tissues and the changes in fish gut microbiota, this study reveals the potential impacts of metal pollution on aquatic ecosystems and fish health. Results indicate that metal pollution caused by mining activities exhibits a distinct spatial distribution in surface water, and the accumulation of metals and metalloids in fish tissues is closely related to pollution levels. Moreover, metal pollution significantly alters the structure and function of the fish gut microbiota, reducing network complexity and stability. These findings emphasize the ecological risks of metal pollution in aquatic ecosystems and provide a scientific basis for developing effective pollution mitigation strategies. Future research should further explore the long-term impacts of metal pollution on fish gut microbiota and its biomagnification in the food chain.

## Figures and Tables

**Table 1:** Content of metals and metalloids in tissues of *Siniperca chuatsi* collected from water samples of the A, B, and C river basins. Results are presented as mean ± standard deviation (*n* = 11).

Simples	Fish Tiss	Ca (mg/kg)	Mg (mg/kg)	Fe (mg/kg)	Mn (mg/kg)	Zn (μg/kg)	Cu (μg/kg)	Co (μg/kg)	Ni (μg/kg)	Cr (μg/kg)	Cd (μg/kg)	Pb (μg/kg)	As (μg/kg)
A	M	554.4 ± 45.4	224.1 ± 102.3	98.9 ± 59.6	0.6 ± 0.3	26.1 ± 13.1	2047.3 ± 1141.1	111.6 ± 56.3	361.2 ± 88.2	2329.3 ± 743.3	19.9 ± 7.6	76.3 ± 41.1	29.5 ± 10.3
	G	5469.3 ± 1003.3	1003 ± 799.5	269.5 ± 100.1	2.6 ± 1.5	217.5 ± 98.4	10,069.5 ± 4584.6	654.4 ± 109.6	1595.9 ± 897.7	9976.6 ± 541.4	217.7 ± 48.7	754.1 ± 132.1	274.1 ± 105.5
	B	1547.2 ± 633.2	255.6 ± 132.2	100.3 ± 10.6	0.8 ± 0.9	35.6 ± 12.5	3047.6 ± 1023.8	244.1 ± 106.8	455.9 ± 109.5	4544.0 ± 2269.1	35.9 ± 15.4	199.4 ± 56.4	45.4 ± 14.7
B	M	523.3 ± 102.2	229.6 ± 136.6	96.3 ± 56.7	0.8 ± 0.2	19.1 ± 9.1	1999.9 ± 966.6	110.9 ± 89.9	377.5 ± 46.1	2024.2 ± 698.3	18.3 ± 5.5	75.5 ± 57.7	27.3 ± 15.5
	G	5147.5 ± 1235.5	897.7 ± 99.3	274.1 ± 104.1	2.1 ± 1.9	189.5 ± 46.1	9846.5 ± 5557.2	444.2 ± 255.1	1895.2 ± 499.2	10,041.3 ± 3875.2	178.4 ± 88.6	809.8 ± 444.1	277.8 ± 97.2
	B	1672.3 ± 899.4	289.7 ± 98.5	121.5 ± 21.7	0.4 ± 0.3	28.4 ± 4.6	3373.8 ± 1115.4	299.4 ± 111.3	555.5 ± 410.0	3339.2 ± 1544.1	29.3 ± 11.4	211.9 ± 109.4	54.4 ± 11.8
C	M	566.4 ± 122.2	287.7 ± 166.4	88.7 ± 42.7	0.9 ± 0.2	20.6 ± 9.3	1987.7 ± 1044.1	118.7 ± 66.6	354.4 ± 86.4	1995.2 ± 446.2	18.4 ± 5.9	67.6 ± 52.2	24.9 ± 14.2
	G	5191.3 ± 1992.1	1047.1 ± 497.1	214.3 ± 100.9	1.2 ± 0.4	276.5 ± 58.9	8887.5 ± 1118.4	599.3 ± 177.2	1478.2 ± 871.5	10,007.8 ± 888.1	169.8 ± 58.4	688.7 ± 245.4	287.1 ± 97.6
	B	2471.1 ± 985.9	299.5 ± 109.3	141.7 ± 45.3	0.6 ± 0.1	36.8 ± 16.1	4475.5 ± 1567.3	224.5 ± 100.9	447.6 ± 123.7	5442.3 ± 1112.3	54.2 ± 21.0	219.9 ± 105.2	49.6 ± 13.0

M—muscles, G—Gut, B—brain.

## Data Availability

The datasets generated and/or analyzed during the current study are available from the corresponding author upon reasonable request.

## Supplementary Materials

The following supporting information can be downloaded at: https://www.mdpi.com/article/10.3390/jox15040124/s1, Figure S1: Linear discriminant analysis (LDA) of gut microbiomes among different region.; Table S1: Summary of Water Analysis Parameters. Table S2: Terminology used to describe the risk factor $E_{r}^{i}$ and RI as suggested by Hakanson [62]. Table S3: Reference values ($C_{n}^{i}$) and toxicity coefficients ($T_{r}^{i}$) of heavy metals in sediments. Table S4: Physicochemical parameters and metal concentrations in water samples (A, B, C). Results are expressed as mean ± standard deviation (n = 11). Values in red exceed the Class III limits of GB 3838-2002. Table S5: The topological characteristics of gut bacterial co-occurrence networks in different pollution gradients (A, B, C).
