# Supplementary material for: Spatial Gradient Effects of Metal Pollution: Assessing Ecological Risks Through the Lens of Fish Gut Microbiota
Source: J Xenobiot. 2025 Aug 3;15(4):124. doi: 10.3390/jox15040124 (PMC12387212; doi:10.3390/jox15040124)
Supplement: Supplementary file 1 [file jox-15-00124-s001.zip › jox-3661563-supplementary.pdf]

Journal of Xenobiotics

MDPI

Supplementary Materials: Spatial Gradient Effects of Metal Pollution: Assessing Ecological Risks Through the Lens of Fish Gut Microbiota

Jin Wei, Yake Li, Yuanyuan Chen, Qian Lin and Lin Zhang

Table S1. Summary of Water Analysis Parameters.

Parameter	Method	Reference
Turbidity (NTU)	YSI Professional Plus	GB3838-2002[1]
Total Nitrogen (TN)	Alkaline persulfate digestion	Zhang et al. (2020) [2]
Total Phosphorus (TP)	Ammonium molybdate method	Li et al. (2022)[3]
Soluble Reactive Phosphorus (SRP)	Ammonium molybdate method	Li et al. (2022) [3]
Chemical Oxygen Demand (COD(Mn))	Permanganate index method	GB3838-2002[1]
NO^{2-}-N, NO^{3-}-N, PO_4^{3-}, SO_4^{2-}	Ion chromatography	GB3838-2002[1]

Table S2. Terminology used to describe the risk factor E_r^i and RI as suggested by Hakanson[4] .

E_r^i	Potential ecological risk for single regulator	RI	Ecological risk for all factor
$E_r^i < 40$	Low	$R_I < 95$	Low
$40 \le E_r^i < 80$	Moderate	$95 \le R_I < 190$	Moderate
$80 \le E_r^i < 160$	Considerable	$190 \le R_I < 380$	Considerable
$160 \le E_r^i < 320$	High	$R_I \ge 380$	Very high
$E_r^i \ge 320$	Very high		

Table S3. Reference values (C_n^i) and toxicity coefficients (T_r^i) of heavy metals in sediments.

Heavy metals	Ni	Cd	Cu	Cr	Pb	Zn
C_n^i (mg/kg)	37.3	0.2	30.7	86.0	26.7	83.6
T_r^i	5	30	5	2	5	1

Table S4. Physicochemical parameters and metal concentrations in water samples (A, B, C). Results are expressed as mean ± standard deviation (n = 11). Values in red exceed the Class III limits of GB 3838-2002.

	A	B	C	Class III Threshold Value (μg/L)	Detection Limits (μg/L)
EC (μS/cm)	300.9±100.3	475±512.3	550.6±155.6	-	-
pH	6.8±0.1	7.35±0.05	7.8±0.1	6.5-8.5	-
OPR (mV)	280.7±2	195.3±1	210.9±1.5	-	-
OD (mg/L)	7.5±0.1	7.8±0.05	8.5±0.1	>5	-
T (NTU)	10.3±0.6	10.9±0.5	15.2±0.2	5	-

TN (mg/L)	1.1±0.05	1.2±0.02	4.2±0.01	2	-
TP (mg/L)	0.17±0.01	0.09±0.05	0.2±0.02	0.2	-
NH_4 (mg/L)	0.05±0.05	0.15±0.02	0.39±0.01	1	-
NO_3 (mg/L)	3.1±0.5	4.2±0.2	7.2±0.1	20	-
NO_2 (mg/L)	0.02±0.02	0.01±0.01	0.15±0.005	0.1	-
SO_4 (mg/L)	55.1±5	59.3±2	85.4±1	250	-
PO_4 (mg/L)	0.02±0.02	0.05±0.01	0.025±0.005	0.2	-
TDS (mg/L)	330±20	300±10	450±5	-	
COD(Mn) (mg/L)	19.3±5.1	15.5±4.5	23.2±5.6	20	-
Ca (mg/L)	62.4±2	65.3±1	20.7±0.5	-	100
Mg (mg/L)	7.6±0.5	7.2±0.2	5.47±0.1	-	100
Na (mg/L)	10.3±0.5	10.2±0.2	8.2±0.1	-	500
K (mg/L)	3.0±0.1	2.5±0.05	3.0±0.02	-	500
Fe (μg/L)	2535.6±56.2	1873.0±49.1	952.5±59.5	300	0.1
Mn (μg/L)	1363.3±56.0	957.0±29.6	750.9±97.1	100	0.1
Zn (μg/L)	630.6±20.7	476.3±110.3	132.6±45.2	1000	0.1
Cd (μg/L)	2.7±0.1	1.5±0.05	1.7±0.02	5	0.001
Co (μg/L)	3.0±0.1	2.0±0.05	1.0±0.3	-	0.001
Cr (μg/L)	23.5±7.4	11.0±3.1	0.5±0.02	50	0.001
Cu (μg/L)	615.7±63.5	217.0±91.2	9.5±2.1	1000	0.01
Ni (μg/L)	15.2±4.2	13.7±5.1	14.5±0.3	20	0.001
Pb (μg/L)	50.3±4.1	25.2±9.8	1.6±0.5	50	0.001
As (μg/L)	2.6±1.1	1.5±0.7	0.3±0.1	50	0.001

Table S5. The topological characteristics of gut bacterial co-occurrence networks in different pollution gradients (A, B, C).

	A	B	C
nodes	62	72	109
edge	803	221	930
Average Degree	25.903	6.139	17.064
Diameter	5	7	7
clustering coefficient	0.764	0.46	0.591
modularity index	0.183	0.647	0.389
Average Path length	1.842	3.331	2.679

positive	93.2	96.8	98
negative	6.7	3.1	1.9

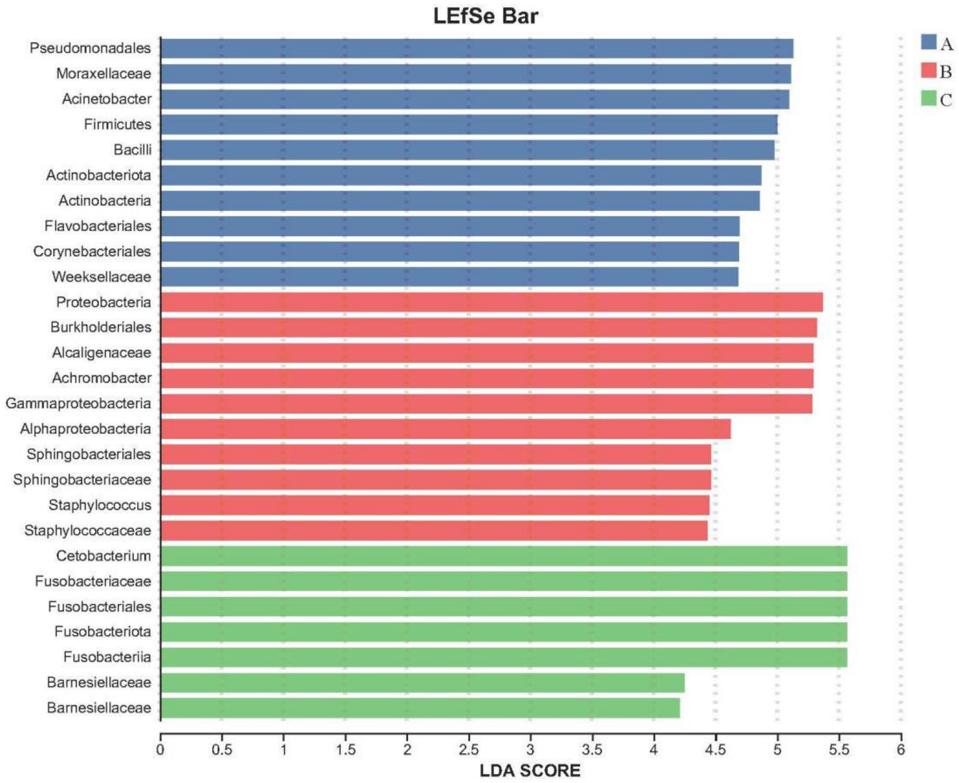

Figure S1. Linear discriminant analysis (LDA) of gut microbiomes among different region.

References

1. GB 3838-2002; Environmental Quality Standard for Surface Water. China Environmental Science Press: Beijing, China, 2002.
2. Zhang Y, Zuo J, Salimova A, Li A, Li L, Li D. Phytoplankton distribution characteristics and its relationship with bacterioplankton in Dianchi Lake. Environmental Science and Pollution Research. 2020 Nov;27:40592-603.
3. Li S, Li B, Liu H, Qi W, Yang Y, Yu G, Qu J. The biogeochemical responses of hyporheic groundwater to the long-run managed aquifer recharge: Linking microbial communities to hydrochemistry and micropollutants. Journal of Hazardous Materials. 2022 Jun 5;431:128587.
4. Hakanson, L., 1980. An ecological risk index for aquatic pollution control.a sedimentological approach. Water Research 14(8), 975-1001. https://doi.org/https://doi.org/10.1016/0043-1354(80)90143-8.
